# A Tragic Tale of Mucormycosis and COVID-19: An Observational Study to Identify Predisposing Risk Factors and Outcomes in a Tertiary Care Centre in Central India

**DOI:** 10.7759/cureus.83479

**Published:** 2025-05-04

**Authors:** Vandana Pandey, Jyotsna Kubre, Tripti Vatsalya, Kamal Ahirwar, Shikha Mehrotra

**Affiliations:** 1 Anaesthesiology, Gandhi Medical College, Bhopal, IND

**Keywords:** covid, diabetes, mucorales, mucormycosis, steroids

## Abstract

Objective

The incidence of mucormycosis, an angioinvasive fungal infection, peaked in India affecting COVID-19 patients. The primary objective of our study was to identify the predisposing risk factors for its development in our country. The secondary objective of our study was to describe the factors affecting the anaesthetic management and the outcome of mucormycosis patients.

Methods

This was a retrospective observational hospital-based study. All the patients with post-COVID mucormycosis planned for surgical debridement in operation theatre were included. Patients with active COVID infection who tested positive by reverse transcriptase polymerase chain reaction, those with cerebral involvement and patients admitted for re-debridement surgeries were excluded from the study.

Results

About 86.6% of patients gave a prior history of COVID infection, while 21.1% did not have any prior history; 77% of patients were diagnosed cases of diabetes, 13% were newly diagnosed and 10% of patients were non-diabetics. About 62.2% of patients gave a positive history of hospitalization whereas 81.1% gave a positive history of use of steroids in some form or the other.

Conclusions

Among all the risk factors, history of COVID-19, presence of diabetes and use of steroids are the most important risk factors which contributed to the development of mucormycosis.

## Introduction

The coronavirus disease 2019 (COVID-19) pandemic swept the whole world in 2020. India reported 44,587,307 cases including 528,629 deaths till January 2023, as per the World Health Organization. India also reported an unprecedented upsurge in mucormycosis cases in patients affected with COVID-19, with almost 51,775 cases on November 29, 2021 [1]. As the world was figuring out the management of COVID-19 disease, a new battlefield of mucormycosis opened up. Mucormycosis is an angioinvasive fungal infection caused by a group of microorganisms belonging to the phylum Glomeromycota [2]. They are ubiquitous, found mainly in soil and decaying organic matter. The incidence rate of mucormycosis globally varies from 0.005 to 1.7 per million population. In India, the prevalence of mucormycosis is estimated as 140 per million population [3]. Following the surge of mucormycosis cases in COVID-19 patients in May 2021, India made it a notifiable disease.

Mucormycoses are life-threatening fungal infections mostly occurring in hematology (acute leukemia or lymphoma), solid organ transplant, or diabetic patients. It may also affect immunocompetent patients following trauma or burn [4,5]. After inhalation of spores in a susceptible individual, it causes complex fungal endothelial interaction leading to vascular invasion and ultimately tissue necrosis. Our centre, an 800-bedded COVID-19 dedicated hospital, experienced a surge of mucormycosis cases while we were dealing with the second wave of COVID-19. Despite the worldwide pandemic of COVID-19, there was an upsurge of cases of mucormycosis from India only [6]. We wanted to find out the factors behind this increase in mucormycosis cases, specifically in India. Thus, we designed an observational study of post-COVID-19 mucormycosis patients with the objective of identifying the predisposing risk factors for its development in our country. The secondary objective of our study was to describe the factors affecting the anaesthetic management and the outcome of mucormycosis patients.

## Materials and methods

This retrospective observational study was conducted in the Department of Anaesthesiology, Gandhi Medical College, Bhopal from May 1, 2021 to May 31, 2021 after institutional ethical committee approval. The requirement of patient consent was waived as it was a retrospective study. All the patients with post-COVID mucormycosis who underwent surgical debridement were included in the study. Patients with active COVID-19 infection who tested positive by reverse transcriptase polymerase chain reaction, those with cerebral involvement and patients admitted for re-debridement surgeries were excluded from the study. Active COVID-19 cases were defined as patients who were laboratory confirmed for SARS-CoV-2 in the fever clinic (by rapid antigen or nucleic acid amplification test). A total of 90 patients were included in the study.

All patients were kept nil per os for 6 hours prior to surgery. In the preoperative area, patients' heart rate, blood pressure, respiratory rate and ambulatory oxygen saturation were noted. The breath-holding time was also noted to assess the pulmonary reserve. The data was collected from the patient's file, preoperative and operative records and surgical OT registers. Data was collected and recorded in MS Excel sheet under the following headings: demographic profile, ASA status, types of mucormycosis, preoperative relevant blood investigations, radiological findings, prior history of COVID-19, history of diabetes, any history of any prolonged illness, history of hospitalization, use of ayurvedic medicines, use of steroids, use of oxygen therapy, use of tocilizumab or remdesivir, preoperative vitals, presence of difficult airway, any intraoperative or postoperative event, whether the patient was extubated, antifungals prescribed - amphotericin or posaconazole - and the outcome of the patient at one week - death or discharge.

After taking informed consent, patients were taken to the operation theatre. Preoperative vitals including heart rate, systolic and diastolic blood pressure, respiratory rate and oxygen saturation were noted. A wide bore intravenous cannula [18G/20G] was secured in the upper limb and intravenous fluid (normal saline/Ringer's lactate) was started. Difficult airway cart and emergency drugs were kept. After adequate preoxygenation, premedication was given which included midazolam, glycopyrrolate and ondansetron intravenously. The patients were induced with fentanyl and propofol. The patient’s airway was secured with a cuffed endotracheal tube after giving the intubating dose of atracurium intravenously. The position of the tube was confirmed by capnography and bilateral chest field auscultation. The tube was fixed at the left angle of the mouth, and sterile throat packing was also done. Patients were maintained on oxygen, nitrous oxide with isoflurane and atracurium. Endoscopic debridement of the necrosed tissue was done using a microdebrider. After completion of surgery, gentle laryngoscopic-guided suction was done, and the throat pack was removed. Patients were extubated after giving a reversal agent on resumption of spontaneous breaths. Patients were subsequently shifted to the intensive care unit for further monitoring and management including intravenous antifungal agents.

Statistical analysis

To summarize categorical data, counts and percentages were used. For normally distributed data, mean and standard deviation were used, and for continuous data, median, range and interquartile range were used. Since it was a retrospective observational study, no analytical tests were used. All the data was analysed using Epi Info software.

## Results

**Figure 1:**
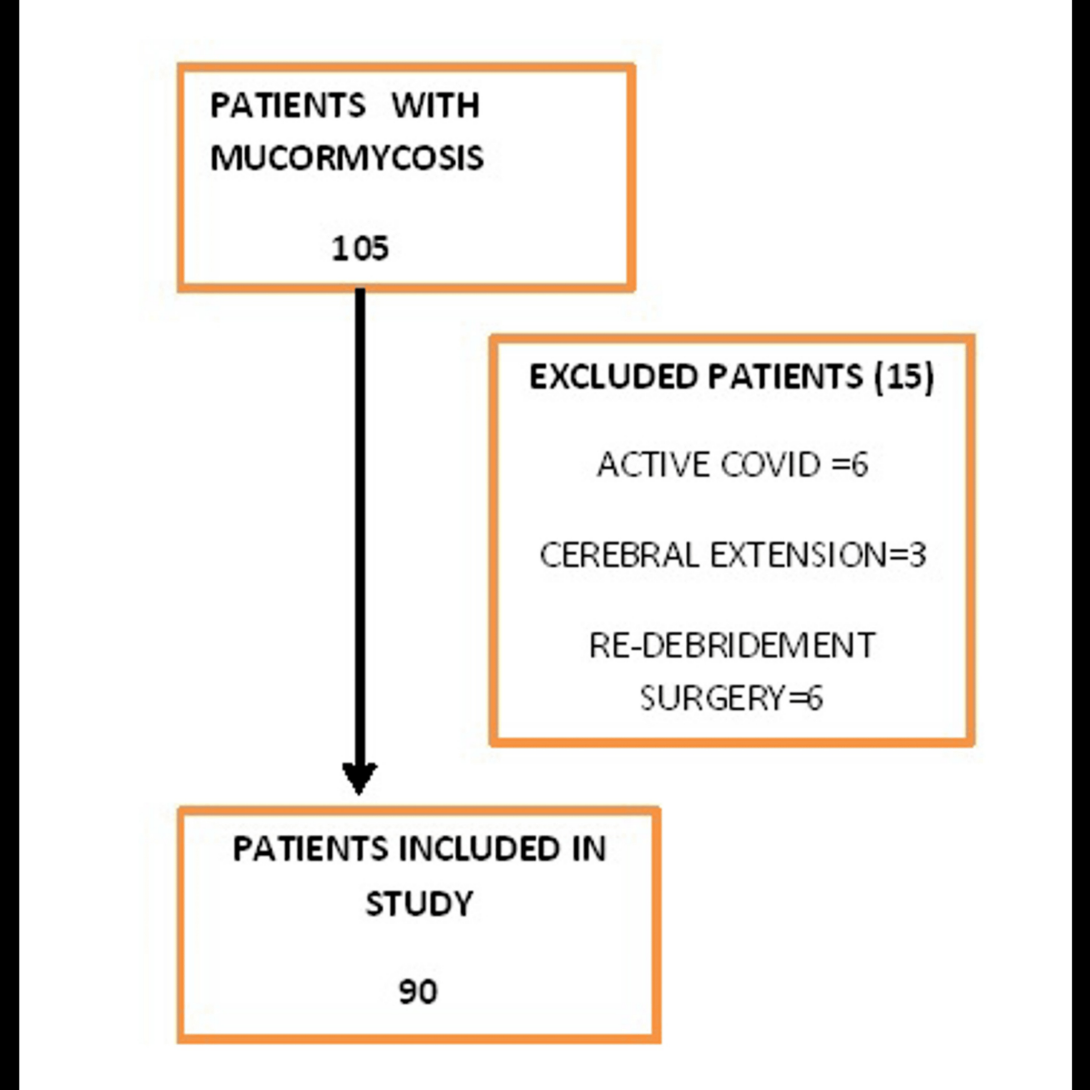
Selection of patients

A total of 105 patients were admitted in our hospital from May 1, 2021 to May 31, 2021. Out of these, 15 patients were excluded from the study as they either had active COVID-19 infection, cerebral involvement or were admitted for re-debridement surgeries (Figure 1). The data of 90 patients who fit the inclusion criteria was collected.

The demographic details and patient characteristics of all patients have been given in Table 1. The mean age of the patients was 51.24±13.1 years. The mean weight of the patients was 67.7±10.2 kilograms. About 67.7% of patients were males. Out of 90 patients, the majority of patients were male and belonged to the American Society of Anesthesiologists (ASA) Physical Status II. About 86.6% of patients gave a prior history of COVID-19 infection while 21.1% did not have any prior history.

**Table 1:** Demographic details ASA: American Society of Anesthesiologists

DEMOGRAPHIC DETAILS (n=90)	MEAN ± SD
AGE	51.24 ± 13.1 years
WEIGHT	67.7 ± 10.2 kilograms
SEX (MALE/FEMALE)	61/29
ASA II/III/IV	69/20/1
HISTORY OF COVID INFECTION	71

**Figure 2:**
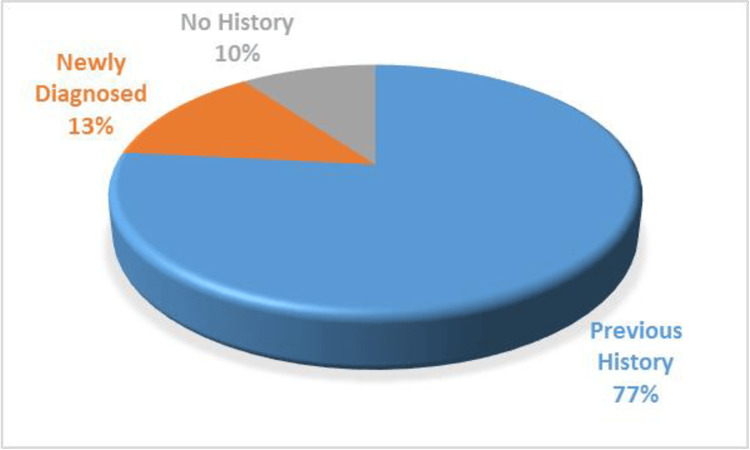
Distribution of diabetes in mucormycosis patients

About 77% of patients were diagnosed cases of diabetes, 13% were newly diagnosed and 10% of patients were non-diabetics as depicted in Figure 2.

About 72.5% of patients had no history of any other prolonged illness or comorbidity, and 16.6% of patients reported hypertension as a comorbid illness. The distribution of other co-morbid illnesses is depicted as a bar diagram in Figure 2.

**Figure 3:**
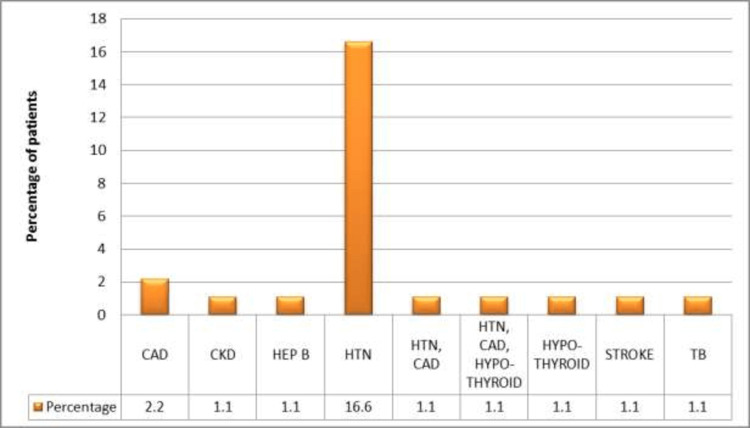
Distribution of comorbidities other than diabetes CAD: Coronary heart disease; CKD: Chronic kidney disease; HEP-B: Hepatitis B; HTN: Hypertension; TB: Tuberculosis

The mean of preoperative investigations like baseline haemoglobin, total leukocyte count, platelet count, fasting blood sugar levels, glycated haemoglobin level (Hb1ac), serum creatinine and serum potassium levels are tabulated in Table 2.

**Table 2:** Details of preoperative investigations HB: Haemoglobin; TLC: Total leukocyte count; FBS: Fasting blood sugar; HBA1C: Glycated haemoglobin A1c

	HB (gm%)	TLC (per mm^3^)	PLATELETS (per mm^3^)	FBS (mg/dL)	HBA1C (%)	CREATININE (mg/dL)	SERUM POTASIUM (mEq/L)
MEAN	10.59	9764.88	2.97	148.46	6.93	1.12	4.38
MEDIAN	10.4	8800	2.8	128.5	6.3	0.78	4.07
SD	1.64	7237.25	1.03	55.21	2.46	2.26	3.03

About 62.2% of patients gave a positive history of hospitalization whereas 81.1% gave a positive history of use of steroids in some form or the other. A total of 53 patients received oxygen therapy - either at home or hospital.

Out of 90, only four patients gave a history of use of ayurvedic medicines, 40 patients received remdesivir and none of the patients received tocilizumab. Eleven percent of patients had a difficult airway. When we measured the outcome of the patient in terms of discharge or death at seven days from surgery, we found that only four of 90 patients died, while the rest were discharged.

## Discussion

Mucormycosis is an opportunistic fatal fungal infection caused by fungi from the taxonomic order Mucorales, the commonest organism being Rhizopus oryzae [7]. There are six clinical types of mucor - rhinocerebral (most common), pulmonary, gastrointestinal, cutaneous, isolated renal and disseminated [8]. The most common type of mucormycosis which we encountered was rhino-orbital.

**Figure 4:**
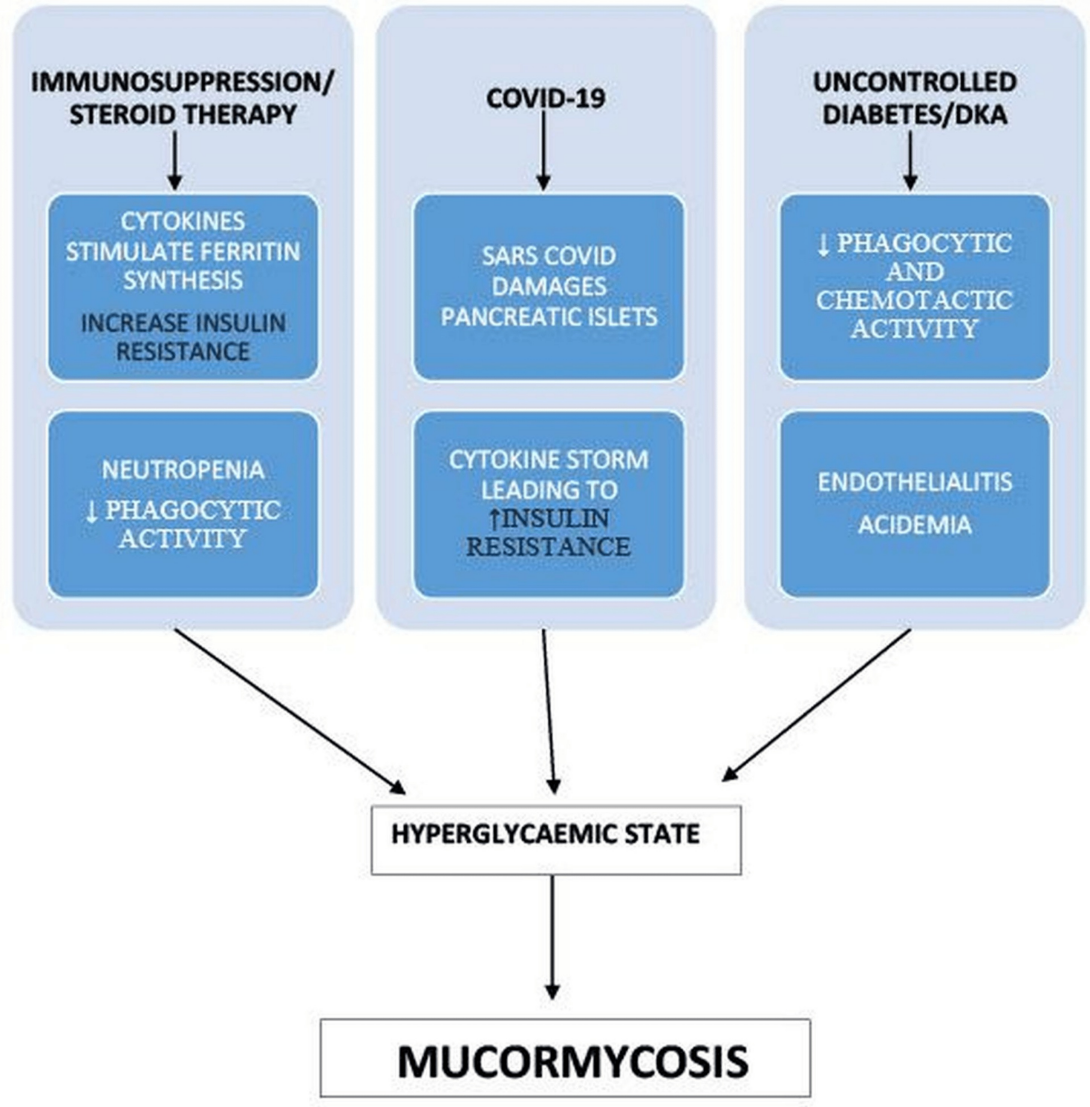
Pathophysiology of mucormycosis in COVID •↑- increase •↓- decrease COVID: Coronavirus disease; DKA: Diabetic ketoacidosis

The cases of mucormycosis increased exponentially following the second wave of COVID-19 in India [9]. The pathogenesis of post-COVID-19 mucormycosis involved a complex synergistic interaction of the predisposing factors like uncontrolled diabetes, use of steroids, and history of COVID-19 [6]. These factors altered the host defense mechanisms, promoted iron uptake by fungi and enabled its binding to endothelial cells leading to widespread ischaemia and tissue necrosis [10]. A schematic representation of this process has been depicted in Figure 4.

Among all the risk factors, a history of diabetes is the most common predisposing factor. Of the total study population, 76.6% of patients were known cases of diabetes mellitus, while 13.3% were newly diagnosed diabetics who presented with elevated blood sugar with mucor for the first time to a health care setting. The newly diagnosed/recently diagnosed diabetic patients were actually diagnosed in the preoperative period. Due to a lack of regular health checkups and unawareness in the Indian population, there were many cases of undiagnosed diabetes which were unmasked by the presentation of mucormycosis in the post-COVID-19 phase. Compliance to anti-diabetic medications is also known to be poor in India [11]. India has 77 million diabetics, which is second only to China in the global diabetic chart and the situation is startling as the predicted diabetic burden in India is 101 million cases by 2030 [12].

There were 19 mucormycosis patients (21.1%) who presented to our hospital without any history of prior infection with COVID-19. Since there was a rise in COVID-19-associated mucormycosis cases, there could be a probability that patients with mucormycosis with no concomitant history of COVID could be either asymptomatic carriers of COVID infection or those who did not get themselves tested. Testing for antibodies against COVID-19 infection would have confirmed whether they had been exposed to COVID-19 or not. Due to the burden of cases and resource limitations, we were unable to do serological testing for antibodies at our institute.

There was no history of any prolonged illness other than COVID-19 or diabetes in 72.5% of patients, which might have acted as a predisposing risk factor for mucormycosis. Only 16.6% of patients had a history of hypertension while there was a low propensity of patients with coronary artery disease, thyroid disorders, chronic kidney disease, stroke, and tuberculosis which are unlikely risk factors for COVID-19.

The rampant use of steroids also contributed to COVID-19-associated mucormycosis [10]. A recent large study suggested that doses and duration beyond the current existing recommendation for COVID-19 (dexamethasone 6 mg up to 10 days) were associated with increased risk of post-COVID mucormycosis [13].

We found that 81.1% of patients had a history of use of inappropriate dose or duration of steroids during the COVID pandemic [14]. In patients infected with COVID-19 infection, the use of glucocorticoids decreases mortality by reducing inflammation-mediated lung injury [15]. The RECOVERY trial provided evidence that treatment with dexamethasone at a dose of 6 mg once daily for up to 10 days reduces 28-day mortality in patients with COVID-19 who are receiving respiratory support. They found no benefit (and the possibility of harm) among patients who did not require oxygen [16].

Higher and prolonged doses caused more harm due to immunosuppression and are a risk factor for invasive fungal infections like mucormycosis. It is defined as a dose of ≥ 0.3 mg/kg corticosteroids for ≥ 3 weeks in the preceding 60 days [17]. In our country, polypharmacy is rampant and steroids are available as over-the-counter medications which can lead to overuse of steroids leading to an increase in secondary opportunistic infections [18]. The use of steroids further caused hyperglycaemia, lymphopenia and immunosuppression which provided a suitable environment for the fungus to thrive [19]. The data on exact dose and duration of the steroid therapy received by patients could not be recovered as the patients were treated at multiple centres by different clinicians during the time of the pandemic. Only 4.5% of patients gave a history of intake of ayurvedic medicines. The role of complementary and alternative medicine in the treatment of COVID-19 has not been established. At most, they may deliver additive treatment along with standard treatment in the form of prophylactic practices and immunomodulation [20].

Another risk factor for mucormycosis, which we investigated, was a history of use of oxygen therapy and hospitalization for COVID-19. We found that 58.8% of patients had received oxygen therapy in some form or the other and 62.2% had been hospitalized. As India was battling the second COVID-19 wave, our healthcare system was overwhelmed. An acute shortage of hospital beds and oxygen, along with limited health care workers, may have led to improper use of oxygen cylinders, use of unclean masks and use of unclean water in the oxygen humidifier. This acted as a growth medium for Mucorales in hospital water [21].

The treatment of mucormycosis involves surgical debridement along with intravenous antifungal drugs. Almost all our patients received antifungal drugs - either amphotericin or posaconazole perioperatively. Anaesthetic management of these patients was challenging as most of these patients were taken up for surgery on an emergency basis without elaborate preoperative blood investigations. We stressed on getting complete blood counts, renal function tests, chest X-ray and blood sugar values for all patients. Patients were assessed in the preoperative room for relevant history and their preoperative vitals were recorded. Airway assessment using standard tools was also made. An estimate of the patients' pulmonary reserve was made on the basis of room air oxygen saturation and breath holding time which guided our further anaesthetic management. These patients have anticipated difficult airway due to facial disfigurement, epiglottitis, and laryngeal edema making both mask ventilation and laryngoscopy difficult. Apart from difficult airway management, these patients also exhibit perioperative haemodynamic instability. This may be due to sepsis, vasodilation secondary to the use of anaesthetic drugs and inflammation. An important cause for hypotension is the prolonged use of steroids in post-COVID-19 mucormycosis-infected patients, which leads to suppression of the hypothalamo-pituitary axis [22]. The use of antifungals like amphotericin B is also associated with many side effects, notably hypokalemia, hypomagnesemia, hypotension and renal insufficiency. The main anaesthetic considerations in post-COVID-19 mucormycosis patients, which we identified at our institute, are summed up in Table 3 [22,23].

**Table 3:** Anaesthetic considerations in post-COVID mucormycosis patients

S.No	ANAESTHETIC CONSIDERATION	DESCRIPTION
1.	Difficult Airway	Difficult mask ventilation due to facial disfigurement; difficult intubation due to epiglottitis and subglottic edema.
2.	Post COVID-19 Sequelae	Immunomodulation; lung fibrosis; myocardial injury; haemodynamic instability; arrhythmia
3.	Diabetes	Reduced host defence mechanisms; hyperglycaemia; endothelialitis and acidemia
4.	Perioperative use of Amphotericin b	Dyselectrolytemia-Hypokalemia, Hypomagnesemia; impairment of renal function; hypotension
5.	Long-term use of steroids	Adrenal suppression causing persistent vasodilatation and hypotension; hyperglycaemia
6.	Need for perioperative blood and blood products transfusion	Due to the extensive surgery, use of anticoagulants/antiplatelets in COVID-19 patients
7.	Need for postoperative ICU care	To manage multisystem involvement of critically ill patients.

All patients were given general anaesthesia using standard institutional protocols. We avoided the use of succinylcholine as its use in post-COVID-19 patients can lead to myopathy and hyperkalemia [24]. We used isoflurane as studies have shown that it halts fungal growth in vitro [25]. The anaesthetic approach to a patient with COVID-19-associated mucormycosis has been depicted in a flowchart pattern in Table 4.

**Table 4:** Approach to anaesthetic management in post-COVID mucormycosis patients

S.No	ANAESTHESIA MANAGEMENT	APPROACH
1.	Preoperative assessment	• Airway assessment • Cardio-Pulmonary reserve assessment • Vitals and baseline investigations • Concurrent illness and medication
2.	Patient counseling and informed consent	
3.	Premedication	
4.	Induction	• Avoid Succinylcholine • Place ETT on left side • Place throat pack • Preferred IV fluids are NS/RL • Hourly RBS monitoring
5.	Maintenance of Anaesthesia	• O2 + nitrous oxide + isoflurane/sevoflurane + muscle relaxant • Standard anaesthesia monitoring • ventilation
6.	Extubation	• Remove throat pack first, suctioning • Reversal agent
7.	Post-operative anaesthesia care unit	• Post operative analgesia • PCM 1g iv TDS and Fentanyl 50mcg iv SOS • Hourly vitals monitoring • Antifungals/antibiotics • Hourly sugar monitoring • Resume early enteral feeding

All patients were handled by a team of doctors from Medicine, Otorhinolaryngology, Ophthalmology, Radiology, Microbiology and Anaesthesiology. Despite the severity of the disease and the presence of predisposing factors, only four patients died at our hospital. The main causes of mortality in these four patients were refractory hypotension due to sepsis and persistent hypoxemia due to severe disease needing ventilatory support. The major limitation of our study was that we could not do a detailed preoperative assessment using pulmonary function tests and echocardiography. Another limitation of our study was that we could not follow up with the patient for the long term. Owing to the burden of the disease at our institute, we could only assess the seven-day mortality of the patients. We also did not include the patients being taken up for re-debridement surgery. Our study also represents the data of a single center. Despite the limitations, our study gives an insight into the mucormycosis burden at a government tertiary care hospital and briefly identifies the predisposing factors leading to the mucormycosis surge amidst the COVID-19 pandemic.

## Conclusions

To conclude, mucormycosis is a fatal opportunistic fungal infection affecting the immunocompromised host. Of all the predisposing factors, history of COVID-19 infection, presence of diabetes or recently diagnosed diabetes and use of steroids were the most important predisposing factors. The perioperative anaesthesia considerations in COVID-19-associated mucormycosis patients need skilful, multipronged management to reduce postoperative mortality and morbidity. Difficult airway, haemodynamic instabilities, dyselectrolytemia, sepsis, perioperative glucose control and postoperative ICU management are some of the anaesthetic challenges faced. Awareness, prevention and prompt treatment of diabetes and standardized prescription policies for the use of steroids are the need of the hour to contain this fatal infection.
